# Optimization and Characterization of Sodium Alginate Beads Providing Extended Release for Antidiabetic Drugs

**DOI:** 10.3390/molecules28196980

**Published:** 2023-10-08

**Authors:** Bence Sipos, Márk Benei, Gábor Katona, Ildikó Csóka

**Affiliations:** Institute of Pharmaceutical Technology and Regulatory Affairs, Faculty of Pharmacy, University of Szeged, Eötvös Street 6, H-6720 Szeged, Hungary; markbenei@gmail.com (M.B.); katona.gabor@szte.hu (G.K.); csoka.ildiko@szte.hu (I.C.)

**Keywords:** nanomedicine, polymeric micelle, antidiabetic, sodium alginate, factorial design

## Abstract

The current research is aimed at investigating the relationship between the formulation components and conditions in the case of a binary drug delivery system, where antidiabetic drugs are co-formulated into polymeric micelles embedded in sodium alginate. Compared to chemical modifications of polymers with alginate, our development provides a simpler and scalable formulation process. Our results prove that a multi-level factorial design-based approach can ensure the development of a value-added polymeric micelle formulation with an average micelle size of 123.6 ± 3.1 nm and a monodisperse size distribution, showing a polydispersity index value of 0.215 ± 0.021. The proper nanoparticles were co-formulated with sodium alginate as a biologically decomposing and safe-to-administer biopolymer. The Box–Behnken factorial design ensured proper design space development, where the optimal sodium alginate bead formulation had a uniform, extended-release drug release mechanism similar to commercially available tablet preparations. The main conclusion is that the rapid-burst-like drug release can be hindered via the embedment of nanocarriers into biopolymeric matrices. The thermally stable formulation also holds the benefit of uniform active substance distribution after freeze-drying.

## 1. Introduction

One of the common denominators of research and development trends in pharmaceutical technology is the aim to optimize formulations with regard to patient needs, clinical needs, and the physicochemical properties of the active ingredient. Among these, the application of nanomedicine is prominent, as is the development of polymeric micelles [1,2]. Polymeric micelles are self-organizing association colloids, the building blocks of which are called amphiphilic graft copolymers. Just like classic surfactants, they can be characterized by the value of the critical micelle concentration and temperature, above which they form nanoparticle-sized (10 to 200 nm) carriers [3,4]. Their utilization brings with it many advantages, as well as increasing water solubility and bioavailability, which is of paramount importance to a significant number of commercialized active substances on the market [5]. Compared to other nanosystems, such as nano-emulsions or lipid-based nanoparticles, it can be said that they are characterized by greater stability and more efficient solubilization [6,7]. However, polymeric systems without surface modification can generally be said to achieve an almost immediate, rapid drug release of the active substance [8].

The rapid release of the active ingredient can be found in many studies and has been proven through different administration routes [9,10]. However, an important question is whether this technological solution is necessary to meet certain therapeutic needs. An excellent example of this is diabetes therapy, where it is important to continuously maintain the appropriate blood sugar level. For this reason, preparations with extended release of active substances dominate the therapy, or active substances with slow metabolism are used, with a long residence time in the blood circulation. In terms of the properties of the active substances, we can find mixed combinations, as both sparingly and highly water-soluble drugs are formulated in the same preparation. A classic example of this is the combination of the highly water-soluble metformin-hydrochloride (MET) and the poorly soluble pioglitazone-hydrochloride (PIO), which is a common combination in second- and third-line therapy protocols for type 2 diabetes mellitus. Nanoparticles have been widely applied in the therapy of diabetes. Novel approaches include the application of nanoparticles with glucose responsiveness to manage insulin levels; however, classic nanoencapsulation technologies can still thrive as tools for the correction and advancement of the physicochemical profile of currently administered drugs [11].

**Figure 1:**
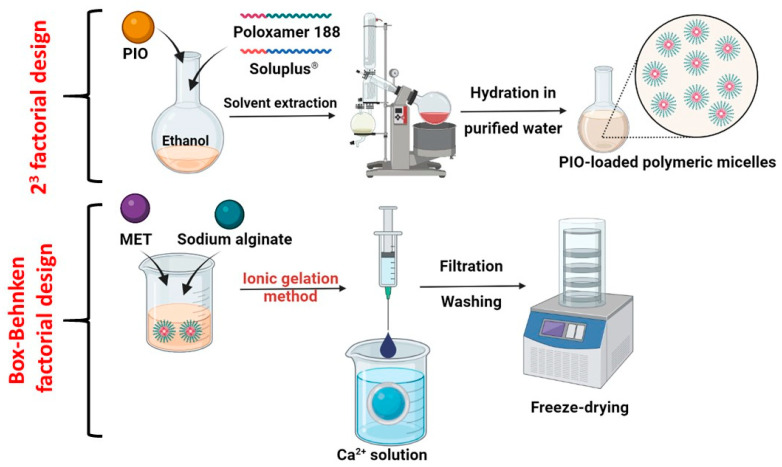
Schematics for the formulation of PIO-loaded polymeric micelles, followed by the incorporation into a sodium alginate bead preparation via the ionic gelation method.

The use of sodium alginate (SA) beads can be an excellent solution to ensure adequate and sustained release. Sodium alginate is the sodium salt of alginic acid, which is a linear polymer composed of repeating units of α-L-guluronic acid and β-D-mannuronic acid. The chemical structure of SA consists of these alternating monomers linked together in a chain-like fashion. It is a biocompatible, water-soluble pharmaceutical excipient utilized for its in situ gelling properties [12,13]. When a solution of SA encounters calcium ions, a crosslinking process occurs: the divalent calcium ions are attracted to the negatively charged carboxylate groups present on the backbone of the alginate polymer [14]. This binding causes the polymer chains to undergo conformational changes, leading to a transition from a sol state to a gel state [15]. With constant contact, the calcium ions continue to bind to the carboxylate groups, and they bridge adjacent alginate chains, creating a three-dimensional network structure, the so-called “egg-box” model [16]. If we can dissolve solubilized nanoparticles alongside the SA matrix, the polymer erosion in the gastrointestinal tract will lead to an extended-release profile of the encapsulated drugs. With the coacervation method, we add the SA solution dropwise to the calcium ion solution, and small beads will be formed; these can be freeze-dried, providing long-term stability and excellent swelling ability, with systems that float on top of gastric juice [17,18] (Figure 1).

This methodology provides physical encapsulation of the drugs and the nanocarrier system; however, there are alternatives where chemical conjugation would occur between the nanocarrier-forming polymer, lipid, etc., and the sodium alginate biopolymer. This, however, influences the critical micellar concentration and other features of polymeric micelles, which might lead to a decrease of encapsulation efficiency, etc. [19,20]. Other bio- or semisynthetic materials include the application of chitosan, zein, or gelatin. Each has its own advantages, but sodium alginate might be the safest option regarding indifferent drug delivery whilst not changing the chemical structure of the nanocarrier system. Current pharmaceutical trends also include special needs such as the elimination of animal-based materials, such as gelatin [21,22,23].

The utilization of polymeric micelles over other carriers lies in their stability as well as their high solubilization profile. Gastrointestinal track conditions include a wide array of different pH levels, osmolality, and transit times, which requires nanocarriers that can withstand these conditions. Liposomes, for example, without any additional excipients degrade rapidly in the gastric media, and the same can be said for protein-based drug delivery systems. Polymeric micelles, especially ones with poly(ethylene-glycol)-based chains, can fit this criterion.

In this research, we aimed to develop a model system where the binary combination of MET and PIO, a common combination in antidiabetic therapy, is embedded inside an alginate bead system. One batch of beads together would contain the low-dose combination, meaning 500 mg of MET and 12.5 mg of PIO. As PIO is a poorly water-soluble drug, at first, it was solubilized via polymeric micelle formation, and following that it was co-formulated with MET inside the SA polymeric matrix. Our hypothesis is that the burst-like drug release effect of polymeric micelles can be hindered and obstructed via incorporation into an SA bead system; to prove this theorem, multi-level factorial design-based optimization and in vitro gastric drug release studies were executed.

## 2. Results

### 2.1. Optimization and Characterization of PIO-Loaded Polymeric Micelles

At first, the polymeric micelle solution containing the antidiabetic drugs must be been optimized. Since MET is a water-soluble drug, it would not incorporate into the core of the micellar structure; however, PIO is a poorly soluble drug, hence it can be solubilized. As mentioned previously, the optimization was performed on the basis that the polymeric micelle forming the polymer composition had the highest impact on the micelle size and micelle size distribution as key indicators of a nanoparticulate system. After preliminary experiments and experience with these polymers, a nine-run 2^3^ factorial design was implemented and executed (Table 1).

**Figure 2:**
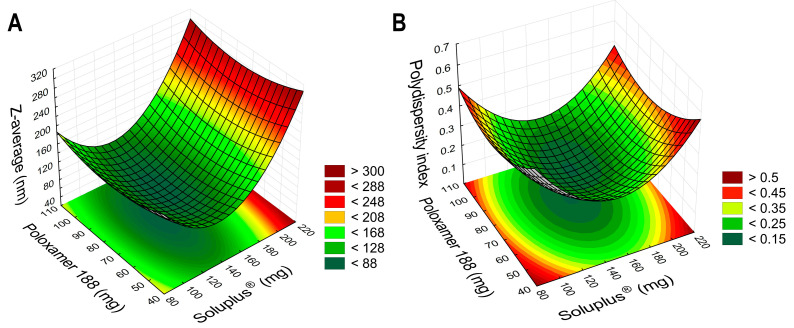
Three-dimensional surface plots based on the results of the 2^3^ factorial design, aiming to optimize the PIO-loaded polymeric micelles. Plot (**A**) describes the relation between the independent factors in the case of the micelle size, as the Z-average; plot (**B**) shows the micelle size distribution, as the polydispersity index.

Based on the results of the dynamic light-scattering measurements, it can be concluded that the choice for the polymer composition was a success, as the data highly varies, most specifically in case of the PdI values. Polymeric micelles are generally below 200 nm in particle size, and some formulations exceeded this criterion. The PdI is also indicative of the success of the formulation if the value is below 0.300; in this case, a monodisperse micelle size distribution is achieved, leading to uniform behavior in colloidal state and after administration. The design spaces were plotted using the TIBCO Statistica^®^ 13.4 Software, and 3D surface plots were generated (Figure 2).

In Equation (1), the second-order polynomial equation can be seen for the Z-average value. The negative coefficients (x_1_^2^, x^2^, and x_2_^2^) mean that by increasing the value of these factors, the micelle size will be decreased, which is beneficial, whilst by increasing the positive coefficient factor x_1_, the size would be increased. The regression coefficient (R^2^) of the surface plots and the constructed polynomial model was 0.9636, and the adjusted regression coefficient (R_adj_^2^) was 0.9372, indicating proper correlation. The only significant factor was the concentration of SP (x_1_) on a linear basis, meaning that the concentration should be lower than the determined maximum (200 mg, as the value for the +1 level of factorial design) to achieve nanosized particles.

(1) $$Z-\mathrm{average}=143.58+39.97x_{1}-32.88x_{1}^{2}-2.95x_{2}-6.96x_{2}^{2}$$

In Equation (2), the second-order polynomial model equation is given for the polydispersity index. All factors had a negative coefficient; however, based on the ANOVA analysis, no factor was significant. The R^2^ and the R_adj_^2^ values were 0.9241 and 0.9114, respectively.

(2) $$\mathrm{PdI}=0.280-0.004x_{1}-0.068x_{1}^{2}-0.032x_{2}-0.029x_{2}^{2}$$

Keeping in mind the significance of each factor and the constructed 3D contour plots, the following composition was chosen: 140 mg of SP and 90 mg of Poloxamer 188 was dissolved in the ethanolic PIO solution, and the formulation process was executed. To test the validity of the results from the factorial design, the polymeric micelles were formulated in triplicate with these amounts of polymers.

**Figure 3:**
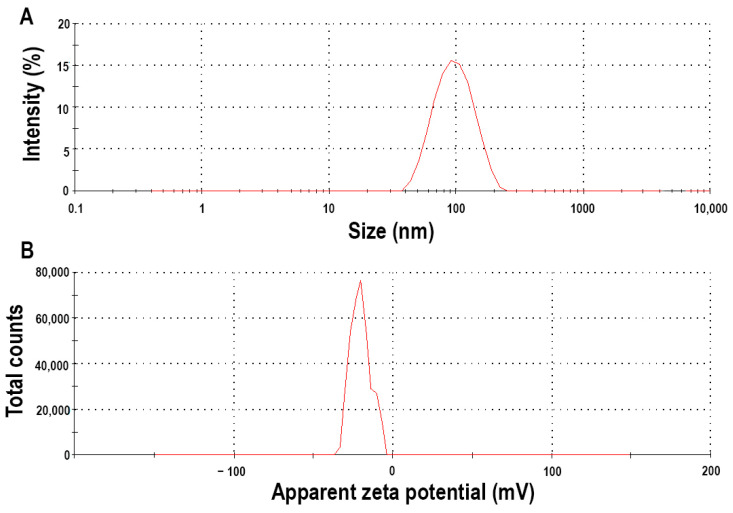
Micelle size (expressed as Z-average) (**A**) and zeta potential (**B**) of the optimized micellar formulation measured via dynamic light scattering.

The Z-average value was 123.6 ± 3.1 nm, indicating that a proper nanoparticulate system was developed, reflecting the average polymeric micelle size (Figure 3). The polydispersity index was 0.215 ± 0.021, which meets the criterion that the formulation should have a monodisperse micelle size distribution. This uniform distribution also allows homogenous and uniform drug release and permeability across epithelial barriers. The zeta potential value was also measured, which was—38.17 ± 4.3 mV, a relatively high value. This high value means that the particles have high repelling forces amongst them, and colloidal stability is provided without aggregation and size increase. The negative coefficient also predicts the route of absorption. Previously, it was found that the polymeric micelles with negative zeta potential value will be absorbed via transcytosis or paracellular transport; these are called mucopenetrating micelles [24].

**Figure 4:**
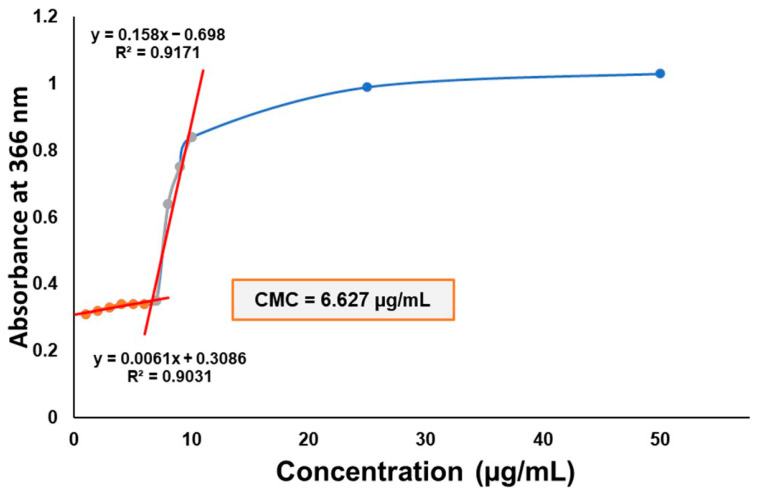
CMC determination of the Soluplus–Poloxamer 188 system measured via iodine UV-Vis spectroscopy. The absorbance at 366 nm is depicted as a function of the concentration of the blank micellar solutions.

The critical micellar concentration (CMC) of the polymeric micelle composition was determined via iodine UV-Vis spectroscopy (Figure 4). The CMC value of Soluplus^®^ (SP), which is 7.6 µg/mL, decreased via the addition of Poloxamer 188 to the system. As the main micelle-forming agent, SP plays the primary role in encapsulating the active substance, providing its solubilization effect; however, the addition of other solubilizers in the form of mixed micellar systems have previously been proven to be useful. The system’s CMC value is approximately 6.627 µg/mL, which is close to its original value of SP; thus, high CMC value decrease is not achieved, i.e., reduction by multiple degrees.

Solubility enhancement–related attributes, such as the encapsulation efficiency and thermodynamic solubility, also reflect on the success of encapsulation. The measured encapsulation efficiency of the optimized formulation was 91.75 ± 3.87%. This is also a high value, meaning that PIO should be encapsulated into the micellar core rather than being in a less-dissolved form or in the form of undissolved particles. The measured solubility of PIO was 0.072 ± 0.005 mg/mL, which was increased to 8.14 ± 0.09 mg/mL in the optimized formulation. These results are also corroborated by the high encapsulation efficiency, the nanoparticle size, and the uniform size distribution.

### 2.2. Optimization and Characterization of Sodium Alginate Beads

To optimize the SA bead formulation loaded with metformin-hydrochloride and the pioglitazone-hydrochloride-encapsulated polymeric micelle, a Box–Behnken factorial design was used. The criteria were that the drug release at the eighth hour should not exceed 70% of the dissolved drugs, and the drug release curve should also show steady growth without spikes. The results from the 15-run trial can be seen in Table 2.

**Figure 5:**
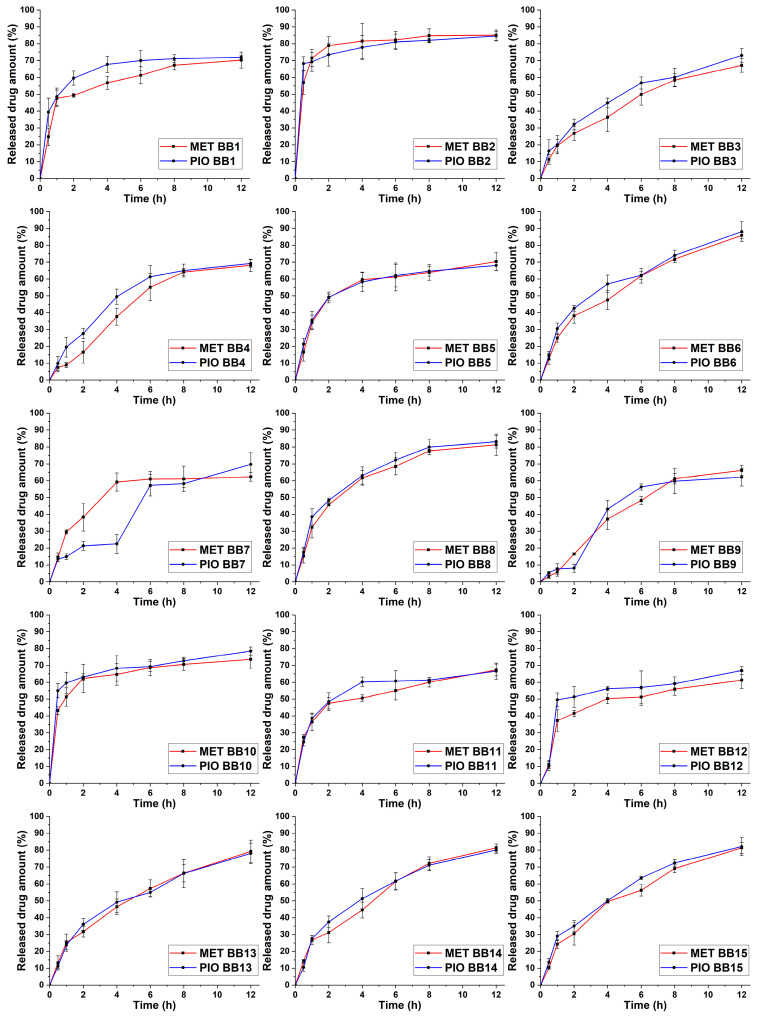
Released drug amount expressed as percentage vs. time drug-release curves in simulated gastric media of the 15-run, 3-factor, 3-level Box–Behnken (BB) factorial design. The number of the trial run can be found in the legend of each curve. Data are presented as average ± SD (*n* = 3).

**Figure 6:**
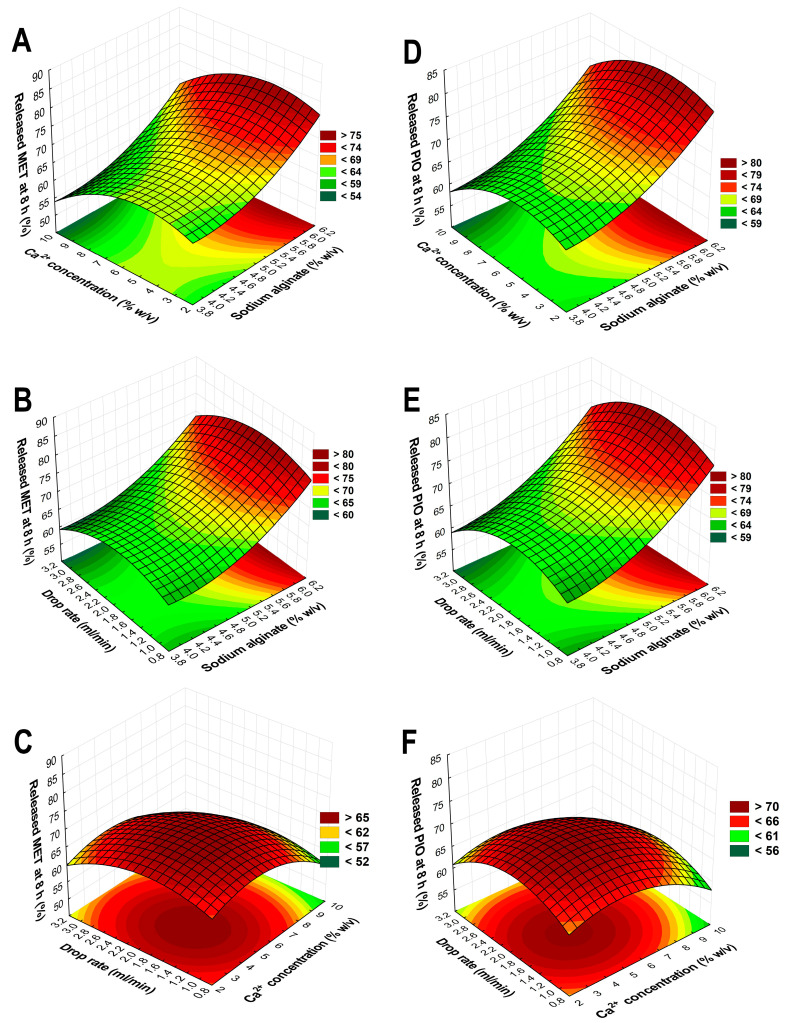
Three-dimensional surface plots based on the results of the Box–Behnken factorial design aiming to optimize the sodium alginate beads. Plots (**A**–**C**) describe the relations between the independent factors in the case of the drug release of metformin-hydrochloride; plots (**D**–**F**) show the drug release of pioglitazone-hydrochloride.

The selection of the independent factors as adjusted variables proves that a proper selection of these were implemented based on preliminary experiments. These results range between 58.37 and 84.76%, also indicating the proper selection. Based on the drug release curves (Figure 5), only a few trial runs meet the criterion of a steady growth; to better describe the relations, 3D surface plots were constructed based on the average of the released drug concentration in the eighth hour time point (Figure 6).

Equation (3) describes the second-order polynomial model regarding the relation between the independent factors and the drug release of MET in in vitro gastric conditions. The R^2^ and R_adj_^2^ values were 0.9315 and 0.9257, respectively, showing proper correlation. Equation (4) reflects the same principle for the drug release of PIO. The R^2^ and R_adj_^2^ values were 0.9458 and 0.9184, respectively, also showing proper correlation. In both cases, the only significant (*p* < 0.05) factor based on the ANOVA analysis is the concentration of SA (x_1_). The similarity between the equations can be explained by the drug release curves in Figure 5, where it can be clearly seen that in most cases, the drug release curves of MET and PIO align with each other, reflecting the drug release modification of the sodium alginate beads.

(3) $$\begin{array}{l} \mathrm{Released}\;\mathrm{MET}\;\mathrm{at}\;8\;h\;\left(\%\right)\\ \;\;\;\;\;\;\;\;\;\;\;\;\;\;\;\;\;\;\;\;\;\;\;\;\;\;\;\;\;\;\;\;=66.40+5.98x_{1}-1.52x_{1}^{2}-3.02x_{2}+1.80x_{2}^{2}-1.60x_{3}\\ \;\;\;\;\;\;\;\;\;\;\;\;\;\;\;\;\;\;\;\;\;\;\;\;\;\;\;\;\;\;\;\;+1.81x_{3}^{2} \end{array}$$

(4) $$\begin{array}{l} \mathrm{Released}\;\mathrm{PIO}\;\mathrm{at}\;8\;h\;\left(\%\right)\\ \;\;\;\;\;\;\;\;\;\;\;\;\;\;\;\;\;\;\;\;\;\;\;\;\;\;\;\;\;\;\;\;=67.09+6.23x_{1}-1.23x_{1}^{2}-2.16x_{2}+1.39x_{2}^{2}-1.19x_{3}\\ \;\;\;\;\;\;\;\;\;\;\;\;\;\;\;\;\;\;\;\;\;\;\;\;\;\;\;\;\;\;\;\;+1.94x_{3}^{2} \end{array}$$

**Figure 7:**
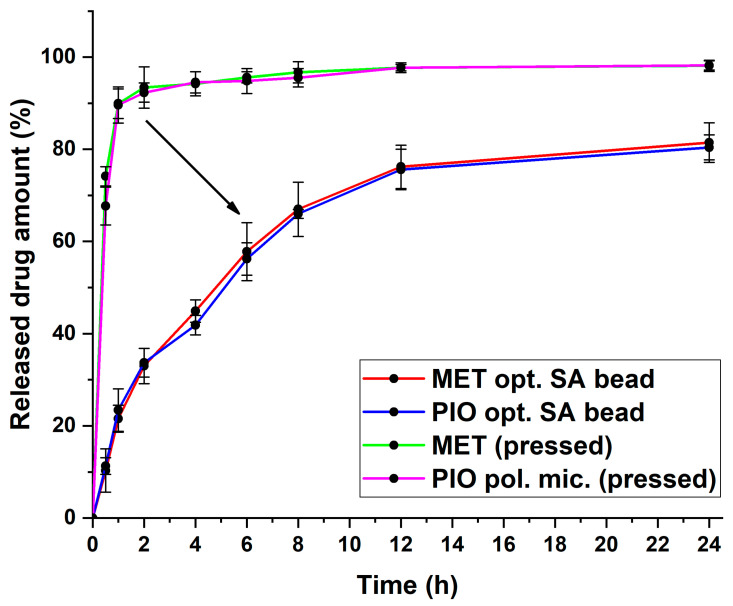
Drug release curves of the optimized sodium alginate bead formulation with the value-added nanoparticulate system. For comparison, pressed powders of MET- and the PIO-loaded polymeric micelles were added. Data are presented as average ± SD (*n* = 3).

Based on Equations (3) and (4), the drug release curves, and the 3D surface plots, the following was concluded: the SA concentration is 5.8% *w*/*v*, and the Ca^2+^ concentration should be 6% *w*/*v*, meaning an almost 1:1 ratio for the gelation process; the flow rate should be 2 mL/min through the pneumatic pump with this syringe setup. Using these determined values during the formulation with the previously optimized polymeric micelle formulation, the optimized formulation had appropriate drug release with a steady growth, and at 8 h, it did not exceed 70% of the released drug amount (Figure 7).

In Table 3, the calculated kinetic parameters can be found. It can be concluded that the dissolved MET would follow first-order kinetics from the SA bead system. However, in the case of the solubilized PIO, the Higuchi kinetics prevailed, with the highest regression coefficient value. The Higuchi kinetics are typical for polymeric micelles, and whilst the time of release is extended in this case, it is similar to those with a rapid, burst-like drug release profile. As can be seen from the drug release curves, the immediate release of the initial substances was slowed down and extended throughout the measurement. This proves the theorem that the classic release-modifying properties of sodium alginate can be utilized to control the release profile of nanoparticles as well.

### 2.3. X-ray Powder Diffraction Study

**Figure 8:**
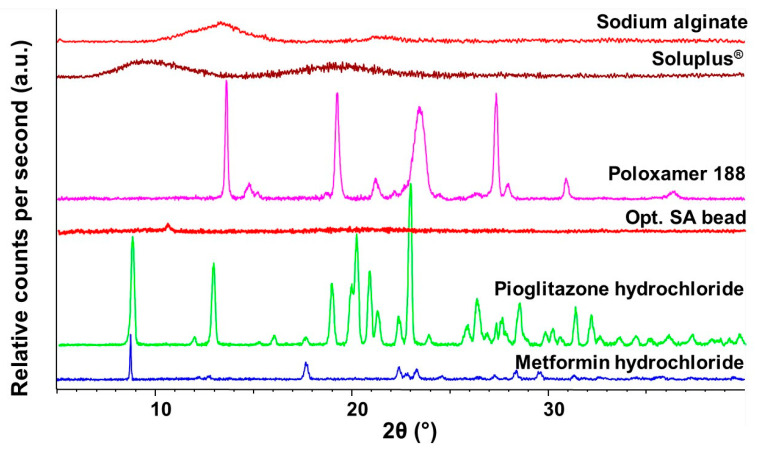
Diffractograms of the initial materials and the optimized, freeze-dried SA bead formulation measured via X-ray Powder Diffraction.

The crystalline structure of the optimized SA bead was performed via X-ray diffraction studies. The initial materials and the final composition were measured (Figure 8). As seen in the diffractograms, the final formulation can be characterized as amorphous, as the characteristic peaks of the two crystalline active substances cannot be detected. Sodium alginate as an excipient is also amorphous; thus, the characteristic peaks are faded in the diffractograms. The amorphous nature proves that the encapsulation was successful; as there is fading of the crystalline peaks.

### 2.4. Thermal Analysis

**Figure 9:**
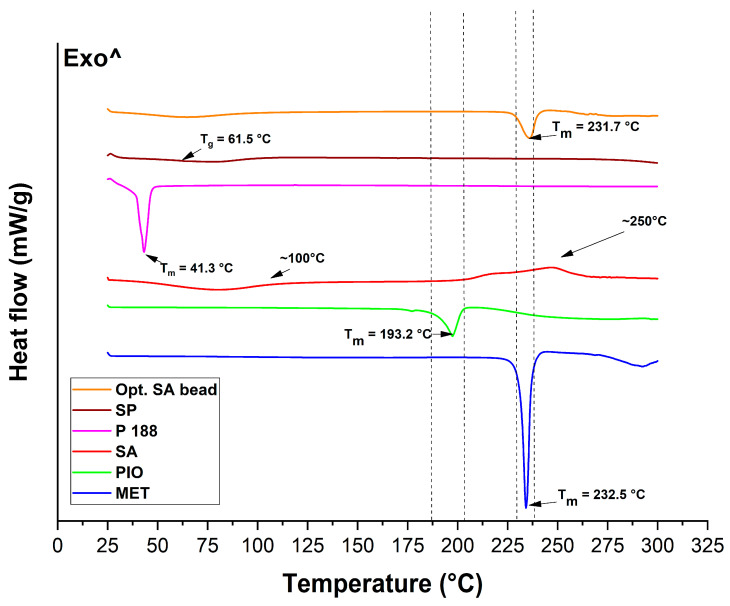
Thermograms of the initial materials and the optimized, freeze-dried SA bead formulation measured via differential scanning calorimetry.

The thermal analysis of the components and the optimized bead formulation was performed via two methods: differential scanning calorimetry (DSC) and thermogravimetry (TGA). The DSC thermograms can be seen in Figure 9. Soluplus^®^ itself is a thermostable co-polymer applied also in melt technology. Thus, no melting points (T_m_) can be detected; only a glass transition temperature (T_g_) of 61.5 °C can be found, which is typical of this polymer. Regarding Poloxamer 188, a sharp endothermic peak can be seen, indicating the melting of the material, most specifically, the poly(ethylene-glycol) (PEG) portion of it. PEGs have a melting point ranging from 3 to 65 °C degrees, depending on the molecular weight. In the case of sodium alginate (SA), two characteristic points can be detected in its thermogram. At first, at around 100 °C, water loss can be found, followed by an exothermic peak (without a sharp peak figure), indicating the degradation process of this biopolymer via heat increase. The two active substances’ melting points can be found also in their thermograms, at 193.2 °C and 232.5 °C for PIO and MET, respectively. The melting point of MET can be found in the optimized SA bead formulation as well, since it has not undergone any encapsulation or chemical modification besides the embedding into the alginate carrier. The melting point of PIO cannot be found in the final formulation, confirming the successful encapsulation into the micellar core.

**Figure 10:**
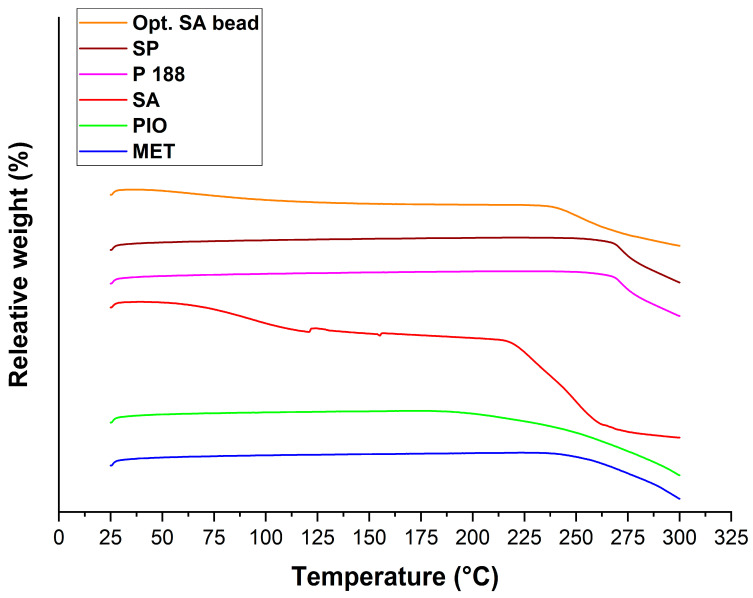
Thermogravimetric analysis of the initial component and the optimized, freeze-dried SA bead formulation.

Thermogravimetric measurements (Figure 10) revealed that the weight loss of the optimized formulation comes from the water loss and the thermal degradation of the sodium alginate, as explained in the DSC evaluation. The total weight loss of SA was as follows: from 25 to 110 °C, 5.6%; from 110 to 275 °C, 49.54%. In case of the optimized formulation, these values decreased to 2.87% and 28.11%, respectively.

### 2.5. Distribution of Active Substances in the Freeze-Dried Sodium Alginate Bead Formulation

**Figure 11:**
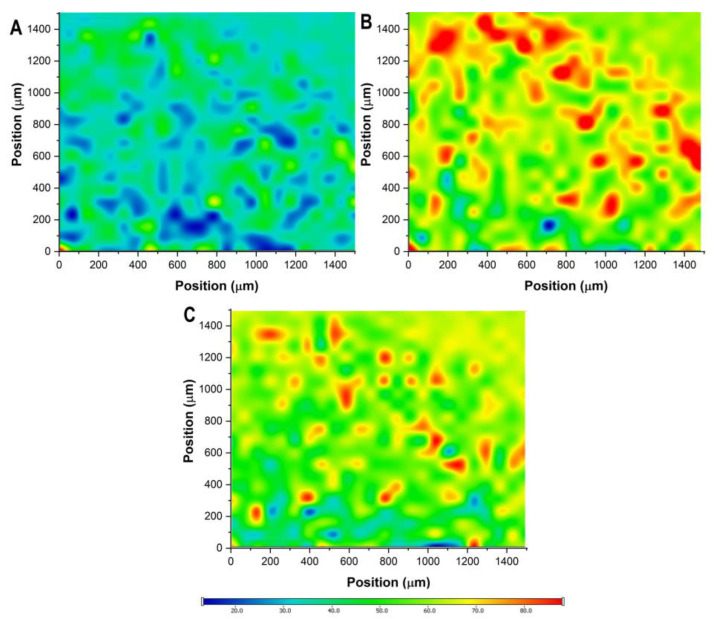
Raman chemical mapping of the freeze-dried optimized sodium alginate bead formulation. Plot (**A**): distribution of pioglitazone hydrochloride; plot (**B**): distribution of metformin hydrochloride; plot (**C**): distribution of sodium alginate.

The distribution of the active substances and sodium alginate was characterized via the cross-sectional Raman chemical map of the freeze-dried formulation (Figure 11). Based on the chemical maps, it can be claimed that the distribution of these substances is quasi-homogenous. The reason behind this could be the freeze-drying process, where the outer layer starts to lose water, followed by the non-uniform shrinking from the outer to the inner layers. The intensity of PIO is relatively low compared to the others, due to the encapsulation process and the difference in quantity.

## 3. Discussion

The utilization of sodium alginate beads can provide many technological pharmaceutical solutions; in this study, we were able to change the characteristics of a nanocarrier to have a different release mechanism compared to conventional forms. Taking therapeutic needs into account is particularly important in the treatment of chronic diseases, including diabetes, for which the technological needs do not necessarily coincide with the value-added nature of innovative nanocarriers. Achieving a sustained release of the active substance is extremely important to achieve when seeking stable blood sugar levels; the use of sodium alginate beads has been proven to be appropriate for this purpose.

In the first step, the value-added polymeric micelle was developed based on the factorial experimental design. Pioglitazone hydrochloride is a poorly water-soluble drug; thus, to match the drug release profile of a highly soluble agent, such as metformin hydrochloride, a solubilization process must be implemented, in this case, in the form of polymeric micelle formation. The micelle size was 123.6 ± 3.1 nm, with a monodisperse size distribution corresponding to a proper polymeric micelle formation. The zeta potential value, indicating colloidal stability, was found to be—38.17 ± 4.3 mV, which is a relatively high value, indicating long-term stability. The encapsulation efficiency was above 90%, which corresponds to the increase in the thermodynamic solubility. Regarding the sodium alginate beads, the optimized formulation provided a stable, long-term extended release and did not exceed the drug release amount of commercialized preparations, which is usually 70% of the total active substance. The Higuchi kinetics prevailed in the case of polymeric micelles, which is typical for these nanocarriers. The structural investigations also confirmed the thermal stability (as per the temperatures of the formulation and the possible application) and the homogenous distribution of the active substances. As a result of the series of experiments, it was proven that conventional active substance-release-modifying systems are able to inhibit the large-scale, burst-type release that is characteristic of polymeric micelles, thereby helping to adapt to the appropriate therapeutic needs.

## 4. Materials and Methods

### 4.1. Materials

MET (N,N-Dimethylimidodicarbonimidic diamide hydrochloride) and PIO ((RS)-5-(4-[2-(5-ethylpyridin-2-yl)ethoxy]benzyl)thiazolidine-2,4-dione hydrochloride) were applied as model drugs in our study and were acquired from Sigma-Aldrich Co. Ltd. (Budapest, Hungary). Soluplus^®^ (SP, poly(vinyl caprolactam)—poly(vinyl acetate)—poly(ethylene glycol, average molecular weight: 90,000–140,000 Da, *f* = 0.43) graft co-polymer (PCL-PVAc-PEG)) was kindly gifted from BASF GmbH (Hannover, Germany), and Poloxamer 188 (P 188, poly(ethylene glycol)-block-poly(propylene glycol)-block-poly(ethylene glycol, average molecular weight: 8600 Da, HLB: 29.0) (PEG-PPG-PEG)) was also acquired from Sigma-Aldrich Co. Ltd. Low viscosity sodium alginate (SA, 4–12 cP (1% in H_2_O, 25 °C; 10,000–600,000 Da)), calcium chloride dihydrate, and chemicals for the in vitro gastric juice (1 g pepsin, 1.5 g mucin ad 8.775 g in 1 l purified water, adjusted with cc. hydrochloride acid to pH 1.2) were also acquired from Sigma-Aldrich Co. Ltd. Analytical-grade solvents ethanol and acetonitrile were purchased from Merck Ltd. (Budapest, Hungary).

### 4.2. Quantification of Metformin Hydrochloride

The quantification of MET was performed via high performance liquid chromatography (HPLC) using an Agilent 1260 (Agilent Technologies, Santa Clara, CA, USA) device. The stationary phase was a Kinetex^®^ C18 column (5 µm, 150 mm × 4.6 mm (Phenomenex, Torrence, CA, USA)). The injection volume was 10 µL, and the eluent flow rate was 1 mL/min. As mobile phases, a 0.02 M PBS (pH 4.5) solution (A) and acetonitrile (B) were applied in a 40-to-60 ratio. Isocratic separation was performed at 25 °C for 3 min. Detection of the chromatograms was carried out at 233 ± 4 nm using a UV-Vis diode array detector. The retention time of MET was 1.21 min. The determined limit of detection (LOD) and quantification (LOQ) were 0.393 ppm and 1.192 ppm, respectively.

### 4.3. Quantification of Pioglitazone Hydrochloride

The quantification of PIO was also performed by HPLC using an Agilent 1260 (Agilent Technologies, Santa Clara, CA, USA) device. The stationary phase was a Kinetex^®^ C18 column (5 µm, 150 mm × 4.6 mm (Phenomenex, Torrence, CA, USA)). The injection volume was 10 µL, and the eluent flow rate was 1 mL/min. As mobile phases, a 0.02 M PBS (pH 5.75) solution (A) and acetonitrile (B) were applied in 50-to-50 ratio. Isocratic separation was performed at 25 °C for 5 min. Detection of the chromatograms was carried out at 280 ± 4 nm using a UV-Vis diode array detector. The retention time of MET was 3.44 min. The determined limit of detection (LOD) and quantification (LOQ) were 0.561 ppm and 1.699 ppm, respectively. All data from the HPLC measurements were evaluated using ChemStation B.04.03 software (Agilent Technologies, Santa Clara, CA, USA).

### 4.4. Formulation of Sodium Alginate Beads

The formulation of the polymeric micelle-embedded sodium alginate beads was performed in multiple steps. First, PIO-loaded polymeric micelles were formulated via the thin-film hydration technique. A total of 12.5 mg of PIO was dissolved in 20 mL of 96% *v*/*v* ethanol alongside the polymeric micelle-forming Soluplus^®^ and the solubilizer Poloxamer 188. The concentration of these excipients varied based on the 2^3^ factorial design. The system was kept under constant stirring (ambient temperature, 200 rpm, 2 h). Subsequently, the solution was transferred into a round-bottom flask, and a Büchi R-210 (Büchi, Flawil, Switzerland) rotation vacuum evaporator was used to extract the solvent; a thin layer of matrix film was formed. The hydration was performed with 20 mL of purified water for 30 min via ultrasonication (Elmasonic S 30 H ultrasonic bath; Elma Schmidbauer GmbH, Singen, Germany). A total of 500 mg of MET was dissolved alongside the formed polymeric micelle solution.

The next step was to formulate the SA beads via the ionic gelation method. The concentration of calcium ions and SA was set based on the optimization via Box–Behnken factorial design. SA was dissolved in the solution of the PIO-loaded polymeric micelles and MET. The measured amount was sprinkled on the surface of the solution, and it was hydrated and completely dissolved using a propeller mixer. A pneumatic pump was used to drop the SA solution into the aqueous solution of calcium chloride. The syringe attached to the pneumatic pump had a diameter of 0.33 mm. The average time for complete addition was 15 min for each batch through this syringe. Upon the gelation, the SA beads were filtered from the calcium chloride solution and rinsed with cold purified water three times. Immediately, the beads were transferred into glass vials (10 to 15 beads per vial) and freeze-dried using a ScanVac CoolSace 100-9 (LaboGene, ApS, Lynge, Denmark) laboratory apparatus. After the freezing, the primary drying was carried out at −40 °C and 0.013 mbar for 12 h, followed by a secondary drying at 25 °C and 0.013 mbar for 4 h.

### 4.5. Optimization of Pioglitazone-Loaded Polymeric Micelles

The optimization of PIO-loaded polymeric micelles was executed via 2^3^ factorial design, where the effect of the amount of the applied polymers on the nanoparticle characteristics (Z-average and polydispersity index) of the polymeric micelles was investigated. The independent variables were investigated at 3 levels, as seen in Table 4.

To investigate the effect of the composition, the quadratic response surface was analyzed, and a second-order polynomial model was constructed using TIBCO Statistica^®^ 13.4 (Statsoft Hungary, Budapest, Hungary). The the relationship of the variables in the response was described via the following second-order equation:

(5) $$Y={\;\beta }_{0}+\beta_{1}x_{1}+\beta_{11}x_{1}^{2}+\beta_{2}x_{2}+\beta_{22}x_{2}^{2}$$

where Y is the response variable; β_0_ is a constant; β_1_ and β_2_ are linear coefficients; β_11_ and β_22_ are quadratic coefficients. Response surface plots for polydispersity index and Z-average in the form of contour plots were plotted according to the regression model by keeping one variable at the center level.

### 4.6. Characterization of Pioglitazone-Loaded Polymeric Micelles

#### 4.6.1. Determination of Critical Micellar Concentration of the Polymer Combination

The critical micellar concentration of the Soluplus^®^–Poloxamer 188 polymeric combination was determined using the iodine UV spectroscopy method. A stock solution of 0.5% *w*/*v* KI/I_2_ was prepared, followed by the preparation of a series of aqueous micellar solutions in varying concentrations. Then, 1 mL of the KI/I_2_ solution was added to each micellar solution, and the solutions were incubated for 2 h at room temperature in the dark. The UV absorbance of varying polymer concentrations at 366 nm was measured using a UV-Visible spectrophotometer (ATI-UNICAM UV/VIS Spectrophotometer, Cambridge, UK).

#### 4.6.2. Measurement of Micelle Size, Size Distribution, and Zeta Potential

The micelle size, expressed as average hydrodynamic diameter (D_H_), and the micelle size distribution, expressed as polydispersity index (PdI), was measured by the means of dynamic light scattering (DLS) via a Malvern Zetasizer Nano ZS (Malvern Instruments, Worcestershire, UK). The zeta potential of the PIO-loaded polymeric micelles was also measured. The measurement took place after the hydration of the PIO-containing polymer film without the addition of MET. The PIO-loaded polymeric micelle solution was measured at 25 °C in folded capillary cells, with the refractive index of 1.640. Each measurement was carried out in triplicate with independent formulations.

#### 4.6.3. Determination of Encapsulation Efficiency

For the determination of the encapsulation efficiency (EE), the PIO-loaded polymeric micelles were separated from the aqueous media via centrifugation after the hydration process using a Hermle Z323 K high-performance refrigerated centrifuge (Hermle AG, Gosheim, Germany) at 10,000 rpm and 4 °C for 45 min. The clear supernatant was diluted 10-fold with 96% *v*/*v* ethanol; then, the quantitative measurements were performed via HPLC [25]. All measurements were carried out in triplicate. The EE was calculated via the following equation:

(6) $$\mathrm{EE}\left(\%\right)=\frac{\mathrm{initial}\;\mathrm{PIO}\;\left(\mathrm{mg}\right)-\mathrm{measured}\;\mathrm{PIO}\;\left(\mathrm{mg}\right)}{\mathrm{initial}\;\mathrm{PIO}\;\left(\mathrm{mg}\right)}\times 100$$

#### 4.6.4. Measurement of Thermodynamic Solubility

To quantify the thermodynamic solubility increase via polymeric micelle formation, 250 µL of purified water was added to the PIO-containing polymeric matrix film. This amount did not dissolve the whole region of the film, and the film was placed in an ultrasonic bath for 6 h. A total of 50 µL of the filtered solution was measured via HPLC. All measurements were carried out in triplicate. The thermodynamic solubility of the initial PIO was measured via the saturation method. A total of 0.5 mL of purified water was measured into a beaker, and an excessive amount of PIO was added. After 6 h of constant stirring (25 °C, 200 rpm) the quasi-suspension was filtered with a membrane filter (pore size: 0.22 µm), and the concentration was measured via HPLC.

### 4.7. Optimmization of Polymeric Micelle-Embedded Sodium Alginate Beads

The optimization of the sodium alginate beads was executed based on the evaluation of a 3-factor, 3-level Box–Behnken factorial design. The independent factors and their values can be seen in Table 5.

As a dependent factor, the released drug amount (expressed as percentage of the initial amount) was chosen. The effect on the drug release was evaluated based on the same principle as in the case of the optimization of PIO-loaded polymeric micelles: a quadratic response surface was analyzed, and a second-order polynomial model was constructed as seen in Equation (7), using the same principle as in Equation (5).

(7) $$Y={\;\beta }_{0}+\beta_{1}x_{1}+\beta_{11}x_{1}^{2}+\beta_{2}x_{2}+\beta_{22}x_{2}^{2}+\beta_{3}x_{3}+\beta_{33}x_{3}^{2}$$

### 4.8. In Vitro Drug Release Study

The modified paddle method (Hanson SR8 Plus (Teledyne Hanson Research, Chatsworth, CA, USA)) was used to determine the drug release profile of the sodium alginate bead formulations in in vitro gastric juice. The paddle was rotated at 100 rpm, the temperature was set at 37.5 °C, and the volume of the dissolution media was 450 mL. The beads were added to the dissolution media; at first, they floated in the media, and then, after a swelling time of 15 to 30 min depending on the variables, they floated on top of it. The sampling was performed at predetermined time points: 0.5, 1, 2, 4, 6, 8, and 12 h. The optimized formulation was also investigated at the 24 h time interval. A total of 1 mL was taken each time as an aliquot for quantification via HPLC. Each experiment was performed in triplicate; the data are presented as average ± SD. Six different mathematical models (zero-order, first-order, second-order, Hixson-Crowell, Higuchi, and Korsmeyer–Peppas model) were fitted with the obtained cumulative drug release vs. time curves to describe the kinetics and calculate the half-time, rate constants, and regression coefficient (R^2^) values [26,27].

### 4.9. X-ray Powder Diffraction Study

To describe the structure of the sodium alginate bead formulation, the X-ray powder diffraction (XRPD) method was used via a Bruker D8 Advance X-ray diffractometer (Bruker AXS GmbH, Karlsruhe, Germany) with Cu K λI radiation (λ = 1.5406 Å) and a VANTEC-1 detector. The applied voltage and amperage were 40 kV and 40 mA, respectively. The angular range was set from 3° to 40° 2θ, with a step time of 0.1 s and a step size of 0.007°. The manipulations and evaluations were carried out using the EVA v4 Software.

### 4.10. Differential Scanning Calorimetric and Thermogravimetric Analysis

Differential scanning calorimetry (DSC) measurement was performed using a METTLER-Toledo 821e DSC (Mettler-Toledo GmbH, Gießen, Germany) at the temperature interval of 25–300 °C and with a heating rate of 10 °C/min under a constant argon flow of 150 mL/min. Every measurement was normalized to sample size and was evaluated with STAR^e^ Excellence Software (https://www.mt.com/sg/en/home/products/Laboratory_Analytics_Browse/TA_Family_Browse/TA_software_browse.html).

Thermogravimetry (TGA) measurements were carried out using a METTLER-Toledo TGA/DSC 1 device (Mettler-Toledo GmbH, Gießen, Germany). A total of 5 ± 0.2 mg of the samples was measured into aluminum pans, closed, and inserted into the furnace. The furnace was heated from 25 °C to 300 °C, with a heating rate of 10 °C/min. The results were evaluated with STAR^e^ Excellence Software.

### 4.11. Raman Spectroscopic Measurement

The Raman spectroscopic measurement was carried out using a Thermo Fisher DXR Dispersive Raman instrument (Thermo Fisher Scientific, Inc., Waltham, MA, USA) equipped with a CCD camera, and a diode laser operating at a wavelength of 780 nm was used. The distribution of PIO, MET, and sodium alginate was investigated by Raman chemical mapping. The freeze-dried bead was cut in half, and the cross-section was analyzed. A 1500 µm × 1500 µm sized surface was analyzed, with step size of 50 µm, an exposure time of 2 s, and acquisition time of 4 s, for a total of 8 scans per spectrum in the spectral range 3500–200 1/cm, with cosmic ray and fluorescence corrections. The Raman spectra were normalized to eliminate the intensity deviation between the measured areas.

### 4.12. Statistical Analysis

Statistical analysis for the measurements and the optimization process were carried out via one-way ANOVA with the post hoc Tukey’s test using TIBCO Statistica^®^ 13.4 (Statsoft Hungary, Budapest, Hungary) software. The analysis of variance (ANOVA) statistical analysis was carried out, and the results were evaluated in harmony with their *p*-value; we considered a variable significant if *p* was less than 0.05 at the 95% confidence level.

## 5. Conclusions

In summary, it can be claimed that the hypothesis was successfully proven: the inhibition of the rapid drug release from polymeric micelles can be performed with traditional matrix-forming systems, as in this case, using sodium alginate. This physical encapsulation method provided the slowing down of the release profile, whilst the main chemical character of the substances remained intact. Furthermore, it was proven that the multi-stage factorial design can help create a polymeric micelle formulation with appropriate nanoparticle characteristics, from which a value-added sodium alginate bead preparation was created. The embedding also resulted in a more thermally stable alginate formulation with an amorphous nature, resulting in proper swelling in the drug dissolution media, mediating the passive diffusion-induced drug release. Based on our results, this preparation could be proposed as an alternative to the extended-release tablets on the market with further considerations.

## Figures and Tables

**Table 1:** Optimization of the PIO-loaded polymeric micelles via a 2^3^ factorial design. The amount of the applied polymers can be seen in the table alongside the results from the dynamic light-scattering measurements: the micelle size represents the Z-average value and the polydispersity index (PdI) represents the micelle size distribution. Data are represented as average ± SD (*n* = 3 from individual batches).

Run No.	Soluplus^®^ (mg)	Poloxamer 188 (mg)	Z-Average (nm)	PdI
1	100	50	165.6 ± 7.4	0.316 ± 0.016
2	100	75	145.7 ± 4.6	0.341 ± 0.022
3	100	75	180.6 ± 3.7	0.440 ± 0.031
4	150	50	143.4 ± 2.2	0.257 ± 0.009
5	150	75	127.4 ± 5.9	0.201 ± 0.010
6	150	100	137.2 ± 8.1	0.345 ± 0.028
7	200	50	287.7 ± 4.1	0.554 ± 0.012
8	200	75	255.2 ± 13.4	0.410 ± 0.046
9	200	100	214.9 ± 2.8	0.276 ± 0.007

**Table 2:** Values of the independent factors and the response-released drug amount expressed as percentage at 8 h under a 15-run, 3-factor, 3-level Box–Behnken factorial design. Data are presented as average ± SD (*n* = 3).

Run No.	SA (% *w*/*v*)	Ca^2+^ (% *w*/*v*)	Drop Rate (mL/min)	Released MET at 8 h (%)	Released PIO at 8 h (%)
1	4.0	3.0	2.0	67.23 ± 2.65	71.14 ± 2.40
2	6.0	3.0	2.0	84.76 ± 4.12	82.02 ± 1.07
3	4.0	9.0	2.0	58.37 ± 3.91	60.11 ± 5.37
4	6.0	9.0	2.0	64.12 ± 2.10	65.01 ± 3.82
5	4.0	6.0	1.0	63.87 ± 4.64	61.65 ± 2.91
6	6.0	6.0	1.0	71.84 ± 2.06	74.02 ± 3.11
7	4.0	6.0	3.0	61.08 ± 7.55	58.26 ± 2.35
8	6.0	6.0	3.0	77.68 ± 0.98	79.94 ± 4.58
9	5.0	3.0	1.0	61.24 ± 3.16	59.70 ± 7.42
10	5.0	9.0	1.0	70.64 ± 3.51	72.79 ± 2.08
11	5.0	3.0	3.0	60.03 ± 2.85	61.28 ± 0.43
12	5.0	9.0	3.0	55.97 ± 3.77	59.15 ± 4.01
13	5.0	6.0	2.0	66.30 ± 5.15	66.12 ± 8.29
14	5.0	6.0	2.0	72.14 ± 3.87	71.09 ± 3.20
15	5.0	6.0	2.0	69.10 ± 2.54	72.42 ± 1.93

**Table 3:** Obtained kinetic parameters of MET- and PIO-loaded polymeric micelles from the optimized sodium alginate beads.

Model		MET	PIO
Zero order	k_0_ (µg h^−1^)	7.8714	7.9351
	R^2^	0.9354	0.9330
	t_0.5_ (h)	6.37	6.30
First order	k_1_ (h^−1^) × 10^−3^	126.3	129.1
	R^2^	0.9890	0.9871
	t_0.5_ (h)	5.49	5.37
Second order	k_2_ (µg^−1^ h^−1^) × 10^−5^	279.0	290.5
	R^2^	0.9684	0.9682
	t_0.5_ (h)	3.85	3.72
Korsmeyer–Peppas	k_K-P_ (h^−n^) × 10^−3^	4.88	4.73
	n	0.5776	0.5667
	R^2^	0.9752	0.9844
	t_0.5_ (h)	5.61	6.40
Higuchi	k_H_ (µg h^−1/2^)	22.805	23.097
	R^2^	0.9643	0.9983
	t_0.5_ (h)	4.67	4.69
Hixon–Crowell	k_H-C_ (µg^1/3^ h^−1^) × 10^−3^	0.1883	0.1926
	R^2^	0.9741	0.9732
	t_0.5_ (h)	5.08	4.97
Best fit		First order	Higuchi

**Table 4:** The investigated polymer amounts at 3 levels in the optimization process of PIO-loaded polymeric micelles.

	Levels		
Independent Factors	−1	0	+1
Soluplus^®^ (mg)	100	150	200
Poloxamer 188 (mg)	50	75	100

**Table 5:** The investigated independent factors at 3 levels in the optimization process of PIO-loaded polymeric micelles.

	Levels		
Independent Factors	−1	0	+1
SA concentration (mg/mL)	4.0	5.0	6.0
Ca^2+^ concentration (mg/mL)	3.0	6.0	9.0
Flow rate (ml/min)	1.0	2.0	3.0

## Data Availability

Data are available on request to the corresponding author.
